# Supplementary material for: Transcriptome Complexity Disentangled: A Regulatory Molecules Approach
Source: Int J Mol Sci. 2025 Mar 11;26(6):2510. doi: 10.3390/ijms26062510 (PMC11942001; doi:10.3390/ijms26062510)
Supplement: Supplementary file 1 [file ijms-26-02510-s001.zip › ijms-3437382-supplementary.pdf]

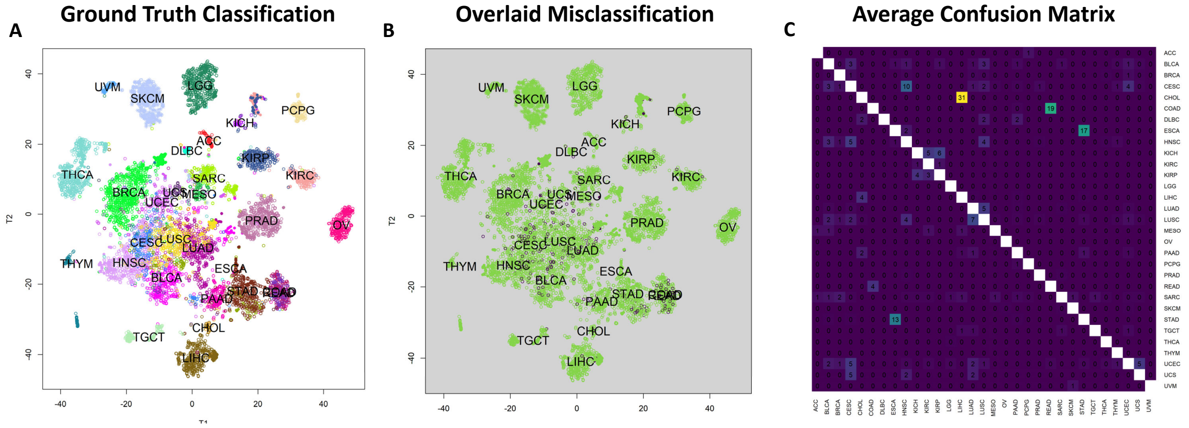

Figure S1. Cancer Type Classification and Visualization Through Regulatory Molecules for TPM normalized TCGA Data. (A) A 2D t-SNE plot of the 8895x47 input TF and miRNA expression matrix, consisting of selected 19 TFs and 28 miRNAs, with colors corresponding to known cancer types, showcasing the ability to differentiate most cancers using 47 regulatory molecules. Note that COAD (Colon Adenocarcinoma) and READ (Rectal Adenocarcinoma) appear almost superimposed due to their high similarity, causing their labels to overlap. **(B)** An enlarged t-SNE plot focusing on misclassified samples, marked in dark purple, indicates that classification errors are more common in highly similar cancer types. **(C)** An average confusion matrix generated from 10-fold cross-validation, exposing the error pattern of the SVM classifier with 90.24% accuracy. The numbers within each cell of the heatmap represent the average percentage of times, across 10-fold cross-validation, that samples from one class (row) were predicted to belong to another class (column). A value of 100% would indicate that all samples of a given class were consistently misclassified as another specific class across all cross-validation folds. Diagonal cells representing correct classifications have been left blank for clarity. Lighter matrix entries (excluding diagonals) represent larger errors, and the error pattern predominantly coincides with the t-SNE visualization of errors in panel B. Classifying similar cancer pairs like STAD and ESCA, COAD and READ, LUSC and LUAD, higher-order similar cancers such as KIRP, KIRC, and KICH, as well as squamous cancers (or cancers with a squamous cell subtype) including LUSC, CESC, HNSC, and BLCA, is challenging. All results are similar to FPKM normalized data with 56 features.

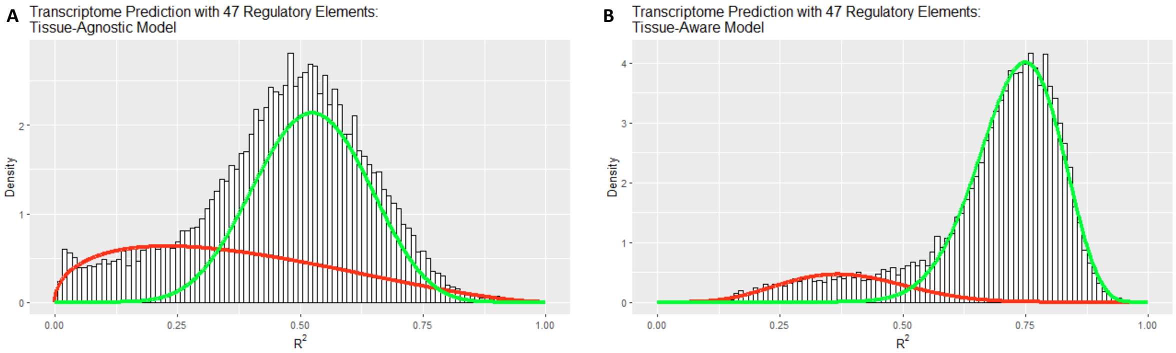

Figure S2. Gene Expression Prediction of Selected Regulatory Molecules with TPM Normalized Data. (A) and **(B)** R^2 histograms for predicting 20,289 gene expressions using 8,895 samples in TCGA with Tissue-Agnostic and Tissue-Aware models, respectively, illustrating the mixture of two components: well-explained genes (green curve) and poorly-explained genes (red curve). The Tissue-Aware model demonstrates enhanced performance, with an average R^2 value of 0.68 compared to 0.46 for the Tissue-Agnostic model.

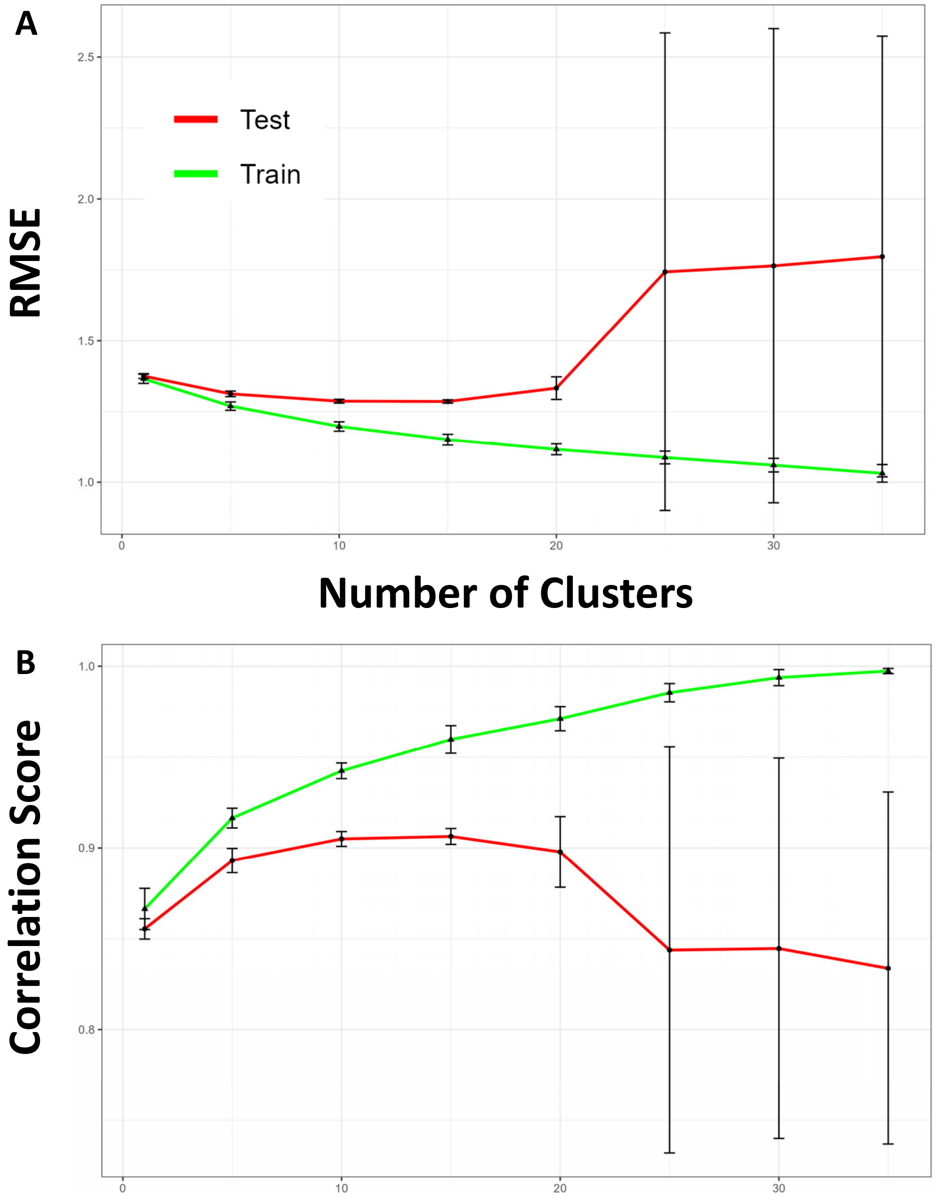

Figure S3. Optimizing Pseudo-Tissues for Tissue-Aware Model Generalization using TCGA TPM Normalized Data. (A) and **(B)** Cross-validation curves for tuning the number of Pseudo-Tissues (sample clusters) based on RMSE and correlation score, respectively, using the TCGA dataset. The optimal number of Pseudo-Tissues is determined to be 15 for both criteria. However, cross-validation assumes that the train and test data share the same distributions, which is not the case when distribution shifts are present, such as when selected miRNA expressions are heavily shifted in CCLE, thus hindering the transportability of the predictive model.
